# Regulation of tumor metastasis and CD8^+^ T cells infiltration by circRNF216/miR-576-5p/ZC3H12C axis in colorectal cancer

**DOI:** 10.1186/s11658-024-00539-z

**Published:** 2024-01-24

**Authors:** Wenqi Du, Xin Quan, Chaoqun Wang, Qiuya Song, Jie Mou, Dongsheng Pei

**Affiliations:** 1 Department of Pathology, Xuzhou Medical University, 209 Tongshan Road, Xuzhou, Jiangsu 221004 People’s Republic of China; 2 School of Pharmacy, Xuzhou Medical University, Xuzhou, China; 3 Department of Human Anatomy, Xuzhou Medical University, Xuzhou, China; 4 Cancer Institute, Xuzhou Medical University, Xuzhou, China

**Keywords:** Tumor immune microenvironment, Metastasis, Colorectal cancer, CircRNF216, MiR-576-5p, ZC3H12C

## Abstract

**Background:**

The tumor immune microenvironment (TIME) is an important regulator of tumor progression, growth and metastasis. In addition, tumor metastasis is one of the principal obstacles to the treatment of colorectal cancer (CRC). Circular RNAs (circRNAs) have been recognized as important regulators in the development of malignancies. However, their specific roles and mechanisms in both CRC metastasis and TIME have not been thoroughly investigated.

**Methods:**

High-throughput next-generation sequencing technology and real-time fluorescence quantitative PCR technology were performed to identify differential circRNAs in CRC. Functional assays including transwell assay, wound healing assay, and metastasis models were conducted to assess the effect of circRNF216 on CRC metastasis. In addition, luciferase reporter, western blot, RNA immunoprecipitation (RIP), and fluorescent in situ hybridization (FISH) were performed to explore the underlying mechanism of circRNF216. The level of immune infiltration was assessed by bioinformatics analysis and flow cytometry in CRC model. Furthermore, rescue and mutation experiments were used for verification.

**Results:**

circRNF216 was identified as a putative tumor suppressor that is downregulated in CRC tissues and cells. Overexpression of circRNF216 inhibits metastasis in vitro and vivo. Mechanistically, circRNF216 acts as a competitive endogenous RNA (ceRNA) for miR-576-5p, alleviating miR-576-5p repression on its target ZC3H12C, which in turn downregulated N-cadherin. Additionally, circRNF216 could enhance the infiltration level of CD8^+^ T cells by upregulating ZC3H12C, ultimately inhibiting the development of CRC, which suggests that circRNF216 is a potential biomarker for the treatment of CRC.

**Conclusions:**

Here, we provide novel mechanistic insight revealing how circRNF216 functioned in CRC metastasis and TIME via the circRNF216/miR-576-5p/ZC3H12C pathway. Therefore, circRNF216 holds promise as a potential therapeutic target and novel diagnostic marker for CRC.

**Supplementary Information:**

The online version contains supplementary material available at 10.1186/s11658-024-00539-z.

## Introduction

Colorectal cancer (CRC) has the third highest incidence rate among cancers, ranking second position among cancers in terms of mortality [1]. The incidence, prevalence, and mortality rates of colorectal cancer remain elevated even in high-income countries [2]. In particular, the 5-year survival rate is less than 10% in those with stage 4 colorectal cancer due to the therapeutic effect of current regimens being limited [3]. Over the past decade, the advent of patient-derived models of CRC, as well as pharmacological and functional assays both in vitro and in vivo, has greatly advanced the development of precision medicine approaches for CRC [4]. Thus, identifying a valuable biomarker and target for therapy for CRC is particularly important.

CircRNAs were first identified in RNA viruses, they were regarded as functionless by products of gene rearrangement and splicing processes [5, 6]. However, as the so-called "byproducts" or "dark matter" of the gene transcription process continued to be discovered, it became clear that many circRNAs were differentially expressed in tumors [7, 8]. Throughout the pre-transcriptional processing of mRNAs by RNA polymerase II, 3′–5′ reverse splicing forms a covalently closed, unbroken loop, resulting in circRNAs [9]. These closed-loop structures are assigned to three classes based on their derivation: exonic circRNAs (EciRNAs), exon–intron circRNAs (EIciRNAs), and intronic circRNAs (CiRNAs) [10]. Numerous studies have demonstrated that circRNAs are rich in miRNA response elements (MREs) and may serve as miRNA sponges [11]. In addition, circRNAs have been documented to play background activities in binding to RNA binding proteins (RBPs) and translating into proteins [12, 13]. Multiple lines of evidence support the intricate involvement of circRNAs in the initiation, relapse, and development of therapeutic resistance in CRC, underscoring their significance as valuable biomarkers and potential therapeutic targets.

The tumor immune microenvironment (TIME) principally consists of distinct immune cell populations in tumor. Tumors interrelated with tumor infiltrating immune cells as well as other immune components through the secretion of various cytokines, such as chemokines, exosomes and other effector molecules, thereby enabling cancer cells to evade surveillance of the immune system [14]. CD8^+^ cytotoxic T lymphocytes (CTLs) are preferred immune cells in TIME for targeting cancer [15]. Therefore, clarifying the mechanisms leading to CD8^+^ T cells infiltration is critical to improve the responsiveness to immunotherapy in CRC. Classical Epithelial-mesenchymal transition (EMT) is a cellular process for embryogenesis, organogenesis, and cancer development [16, 17]. Cells undergoing this process lose epithelial-like characteristics and integrity, acquire the mesenchymal features and become motile [18]. Cadherins play a crucial role in facilitating cell–cell adhesion within epithelial tissues, which is closely linked to tumor migration and invasion. N-cadherin is identified as a cell adhesion molecule expressed by interstitial cells for its role in cell attachment [19], differentiation into specialized tissues, and influencing signaling in various cellular processes [20, 21]. Sufficiently appreciating the relationship of circRNAs and cell adhesion in CRC development will enhance our comprehension of molecular mechanisms and inform the etiology and preventive strategies.

Here, we identified circRNF216 as a specific circRNA downregulated in CRC patients through RNA sequencing (RNA-seq) technology. Corresponding with our study, circ-RNF216 was identified as downregulated in radioresistance extracellular vesicles (RR‑EVs) compared to Nor‑EVs derived from glioma cells [22]. Our findings demonstrate that circRNF216 inhibits CRC metastasis by acting as a sponge for miR-576-5p, thereby upregulating the ZC3H12C level. Additionally, circRNF216 is shown to enhance the infiltration of CD8^+^ T cells and trigger the release of soluble factors. As a result, this study illuminates the tumor-suppressing role of circRNF216 and suggests its potential as a predictive marker for tumor progression in CRC.

## Materials and methods

### Tissue samples

Forty human CRC and adjacent normal tissue samples were obtained from CRC patients intraoperatively at Xuzhou Medical University Affiliated Hospital (Xuzhou, China) between 2014 and 2016. Three paired tumor and adjacent non-carcinoma tissues were applied in RNA-sequencing (RNA-seq). No patient received preoperative radiation therapy or chemotherapy. The tissue samples were immediately frozen in liquid nitrogen prior to use. The median expression level of circRNF16 was determined to classify samples into high or low circRNF216 expression group. The study protocol was approved by the Ethics Committee of Xuzhou Medical University. Clinical specimens were obtained with the informed consent of patients.

### Cell cultures

The CRC cell lines SW620 (Cat. No.: TCHu101), SW480 (Cat. No.: TCHu172), HCT116 (Cat. No.: TCHu99), HT-29 (Cat. No.: TCHu103), DLD1 (Cat. No.: TCHu134) and CT26 (Cat. No.: TCM37) were provided by the Cell Bank of Chinese Academy of Sciences (Shanghai, China). Meanwhile, the immortalized colon epithelial cell line FHC was obtained from the American Type Culture Collection (CRL-1831, ATCC, VA, USA). Thereafter, the DLD-1 and HT-29 cell lines were cultured in RPMI-1640 medium (Bio-Channel, Nanjing, China) supplemented with 10% FBS (ExCell Bio, Shanghai, China), whereas the HCT116, SW620, SW480, and CT26 cell lines were cultivated in DMEM (Bio-Channel, Nanjing, China). All the above cell lines were free from mycoplasma contamination and incubated in the CO_2_ water-jacketed incubator at 37 °C with 5% CO_2_*.*

### Vector construction and cell transfection

The circRNF216 and ZC3H12C sequences were cloned into the pcDNA3.1(+) overexpression vector (IBSBIO, Shanghai, China). As a control, an empty vector (mock) was employed*.* siRNAs specifically targeting circRNF216 were purchased from GenePharma (Shanghai, China). Quantitative real-time PCR (qRT-PCR) and Western-Blot (WB) assays were conducted to evaluate the transfection efficiency. Synthetic miR-576-5p mimics and inhibitor were provided by Gene Pharma (Shanghai, China). Subsequently, Liposomal Transfection Reagent (YEASEN, Shanghai, China) was used to transfect CRC cells with 4 μg of plasmid per plate, while siLentFect Lipid Reagent (Bio-Rad, CA, USA) was employed to deliver 200 pmol of interference fragment per plate. The transfection mixture was incubated for 6 h, after which it was removed and replaced with fresh growth medium containing serum. After 24 h or 48 h, cells were harvested for subsequent analysis.

### RNA and genomic DNA (gDNA) extraction

RNA samples were extracted using Trizol Reagent (Vazyme, Nanjing, China). In brief, 1 ml Trizol reagent was mixed with the sample by vortexing, followed by the addition of 200 μl chloroform. Meanwhile, the whole gDNA was extracted with the TIANamp Genomic DNA Kit (TIANGEN, Beijing, China).

### Quantitative real-time PCR (qRT-PCR)

For qRT-PCR assay, RNA was retrotranscribed with the SweScript RT I First Strand cDNA Synthesis Kit (Servicebio, Wuhan, China). By using 18S rRNA as an internal control, the relative expression of circRNA was calculated by the 2^−ΔΔCT^ method. U6 and GAPDH were used as the endogenous controls for qRT-PCR of miRNA and mRNA, respectively on the StepOnePlus System (ABI, CA, USA). Moreover, the abundances of circRNA and linear mRNA were assessed by divergent primers and convergent primers, respectively. All primers are listed in Additional file 1: Table S1.

### Transwell assay and wound healing assay

In the 24-well transwell plates (pore size, 8-mm; Corning), the migratory and invasive capacities of CRC cells were assessed in vitro. In brief, 1 × 10^5^ cells were cultured with serum-free medium and then seeded in the upper chambers coated with or without Matrigel (BD Biosciences, NJ, USA), while the serum-containing medium was added in the bottom chambers. Migratory capacity of CRC cells was measured after 12 h, while invasive capacity was detected at 24 h later. Thereafter, cells were fixed with 4% paraformaldehyde (PFA) and later stained with 0.1% crystal violet. Finally, cell images were captured on an IX-71 inverted microscope (Olympus, Tokyo, Japan) at 100 × magnification.

The wound healing assay was conducted on the six-well plates, and cells were seeded at a density of 1 × 10^6^/ml. Then, a 200-μl pipette tip was used to make an artificial wound across the cell monolayer. Cell migration was observed and photographed at 0 and 24 h using a microscope.

### WB assay and antibodies

Total proteins extracted from indicated cells were lysed in RIPA lysis buffer (VICMED, Xuzhou, China) supplemented with 1 mM PMSF (VICMED, Xuzhou, China) following manufacturer’s instructions. The BCA Protein Assay kit (KeyGEN, Nanjing, China) was applied in the determination of protein concentration. Proteins were then quantified, separated by SDS*-*PAGE and transferred electrophoretically onto nitrocellulose membranes. Then, membranes were incubated with primary antibodies at 4 °C overnight, including anti-ZC3H12C (1:500, NOVUS, CO, USA), anti-AGO2 (1:50 for RIP, Abcam, UK), anti-N-cadherin (1:1000, Servicebio, Wuhan, China), anti-β-actin (1:5000, Proteintech, IL, USA), and anti-GAPDH (1:5000, Proteintech, IL, USA).

### Luciferase reporter assay

To identify the binding of miR-576-5p to circRNF216 or its target gene ZC3H12C, the 3′-UTR sequences predicted to interact with miR-576-5p, as well as the corresponding mutated sequences within the predicted target site and inserted in the pmirGLO luciferase vector (IBSBIO, Shanghai, China) were constructed and termed circRNF216-WT, circRNF216-MUT, ZC3H12C-WT, and ZC3H12C-MUT, respectively. Subsequently, the above sequences were co-transfected with miR-576-5p mimics in HCT116 cells for detection using the KeyGEN Dual Luciferase Assay Kit following manufacturer's instructions (Nanjing, China).

### RNA immunoprecipitation (RIP) assay

After overnight incubation with anti-AGO2 or control IgG antibody at 4 °C, 0.5 ml lysis buffer was added to incubate the beads for 4 h at 4 °C. Total RNAs were extracted from the magnetic beads using TRIzol (Vazyme, Nanjing, China). After immunoprecipitation, RNAs were prepared into cDNA through reverse transcription for qPCR analysis.

### Fluorescence in situ hybridization (FISH) assay

The Cy3-labeled circRNF216 probes and FAM-labeled miR-576-5p were provided by IBSBIO (Shanghai, China) and Servicebio (Wuhan, China). Hybridization was carried out overnight at 37° using circRNF216 and miR-576-5p tagged probes. Images were acquired by Leica STELLARIS 5 (Leica, Germany).

### Hematoxylin and eosin (H&E) and immunohistochemical (IHC) staining

The tissues were embedded in paraffin, cut into 5-μm-thick sections, and stained with H&E. On the other hand, IHC analysis was conducted on the paraffin-embedded tissues. Then, lung sections were incubated with the indicated anti-ZC3H12C and anti-N-cadherin antibodies overnight at 4 °C. Images were acquired using an Axio Scope A1 upright microscope (Zeiss, Germany). The percentage of positively stained cells was categorized into four groups: Category 1 (0–25%), Category 2 (26–50%), Category 3 (51–75%), and Category 4 (76–100%). The staining intensity was scored on a scale of 0–3 (0 = negative; 1 = weak; 2 = moderate; 3 = strong). The staining level was assessed using the immunoreactive score (IRS), which was calculated by multiplying the scores for the percentage of positive cells and the staining intensity.

### Lentiviral transfection and animal experiments

Virus infection in HCT116 cells was performed using 5 µg/ml polybrene. The 4-week-old female Balb/c nude mice were customized by GemPharmatech (Nanjing, China) and randomized into two groups (*n* = 5 each). For the induction of lung metastases, 5 × 10^6^ cells were resuspended in 200 μl PBS, and the resuspension was later injected into mice via tail vein. Six weeks later, the mice were euthanized, and the lungs were dissected to find the metastatic sites.

To detect tumor infltrating lymphocytes, the stable CT26 cell line cells stably expressing the relevant construct were selected with 2 μg/ml puromycin for 15 days. Stable CT26 cells transfected with vector, circRNF216^OE^, circRNF216^OE^ + ZC3H12C^KD^ were concentrated to 2 × 10^6^/100 μl PBS and subcutaneously injected into the flanks of C57BL/6J mice (*n* = 5). The tumor tissue was digested in a specific medium consisting of 100 ml of DMEM supplemented with 2 ml of FBS, 0.1 g of collagenase I (Solarbio, C8140), 50 μl of hyaluronidase (Solarbio, H8030), and DNase I (Solarbio, D8071) for 30 min at 37 ℃. Cells were stained with respective antibodies (CD3, Thermo Fisher, 47-0031-82; CD8a, Thermo Fisher, 46-0081-82); for 30 min at 4 ℃. All stained cells were measured using FACS Calibur (BD Biosciences, USA). The data were analyzed using the FlowJo software (Treestar Inc., Ashland, OR, USA). All the experimental protocols were authorized by Xuzhou Medical University.

### Isolation of CD8^+^ T cells

Human peripheral blood mononuclear cells (PBMCs) were isolated from healthy individuals using density gradient centrifugation with Ficoll buffer. Subsequently, the CD8^+^ T cells were specifically labeled on their surface with CD8 antibodies. Magnetic beads were then conjugated to these CD8 antibodies, resulting in the formation of CD8 antibody-magnetic bead complexes. Upon applying a magnetic field, the complexes were attracted and immobilized, facilitating their separation from the unbound cells present in the supernatant. The supernatant containing the unbound cells was carefully transferred to a new tube for further analysis. Following the removal of the magnetic field, the CD8^+^ T cells bound to the magnetic beads underwent several washes to eliminate any residual impurities. Finally, the purified CD8^+^ T cells were collected through centrifugation and cultured in in RPMI-1640 culture medium containing 10% FBS, 1% antibiotics, and 10 ng/ml human IL-2, enabling their subsequent use in the T cell proliferation assay.

### T cell proliferation assay

The supernatant culture medium from HCT116 or SW480 cells transfected with vector, circRNF216^OE^, circRNF216^OE^ + ZC3H12C^KD^ for 48 h were plated in the upper chamber. The isolated CD8^+^ T cells (5 × 10^4^ cells) were covered into the lower compartment of transwell chambers. After a 24-h co-incubation, a total of 50 µl of CCK-8 reagent was added to the lower compartment of transwell chambers. The culture dish was then placed in 37 °C incubator for 4 h to facilitate the absorption of the reagent by the cells. Subsequently, the absorbance of each well was measured at a wavelength of 450 nm using a Multiskan Spectrum 1500 (Thermo Labsystems, USA).

### Enzyme-linked immunosorbent assay (ELISA)

Vector, circRNF216^OE^, circRNF216^OE^ + ZC3H12C^KD^ CT26 cells were injected to C57BL/6J to establish subcutaneous implantation tumor models. Then the tumors were collected for ELISA analysis after 18 days. The supernatants were harvested and measured by mouse Perforin1, Granzyme B, IFN-γ, TNF-α ELISA Kit (Elabscience Biotechnology Co., Ltd) according to manufacturer’s instructions.

### Bioinformatics analysis

CircInteractome (https://circinteractome.irp.nia.nih.gov/) and ENCORI (The Encyclopedia of RNA Interactomes, http://starbase.sysu.edu.cn/index.php) were utilized to predict miRNA that more likely to bind with circRNF216. TargetScan (https://www.targetscan.org/vert_80/), miRDB (https://mirdb.org/), and miRTarBase (https://mirtarbase.cuhk.edu.cn/) databases were used to identify the target genes for miR-576-5p. TNM plot (https://tnmplot.com/analysis/) and TIMER (https://cistrome.shinyapps.io/timer/) were used to compare the expression of ZC3H12C and tumor infiltration immune cells in tumor and normal tissues. We searched for ZC3H12C on BioGRID (https://thebiogrid.org) website and then obtained a genes network associated with ZC3H12C. We utilized the HPA database (https://www.proteinatlas.org) to evaluate immunohistochemical staining of ZC3H12C in CRC. Kaplan Meier-plotter (http://www.kmplot.com/analysis/) was used to assess the correlation between ZC3H12C and overall survival rate. The KEGG and GO pathway analysis was generated from LinkedOmics (http://www.linkedomics.org/login.php). Correlation of ZC3H12C and immune effector cells was analyzed by TISIDB website (http://cis.hku.hk/TISIDB).

### Statistical analysis

SPSS 22.0 (SPSS, USA) and Prism (GraphPad Software) were employed for statistical analysis. The significance of differences between the two groups was evaluated by two-tailed Student’s *t*-test or one-way analysis of variance (ANOVA). In addition, the relationship between circRNF216 and clinical parameters was analyzed with Chi-square test. Overall survival (OS) was analyzed according to the Kaplan*–*Meier method and compared by log*-*rank test. The data were expressed as means ± standard deviations (SD). *P* < 0.05 suggested that the differences between groups were statistically significant*.*

## Results

### Identification of circRNF216 via RNA-Seq in CRC tissues

**Fig.1:**
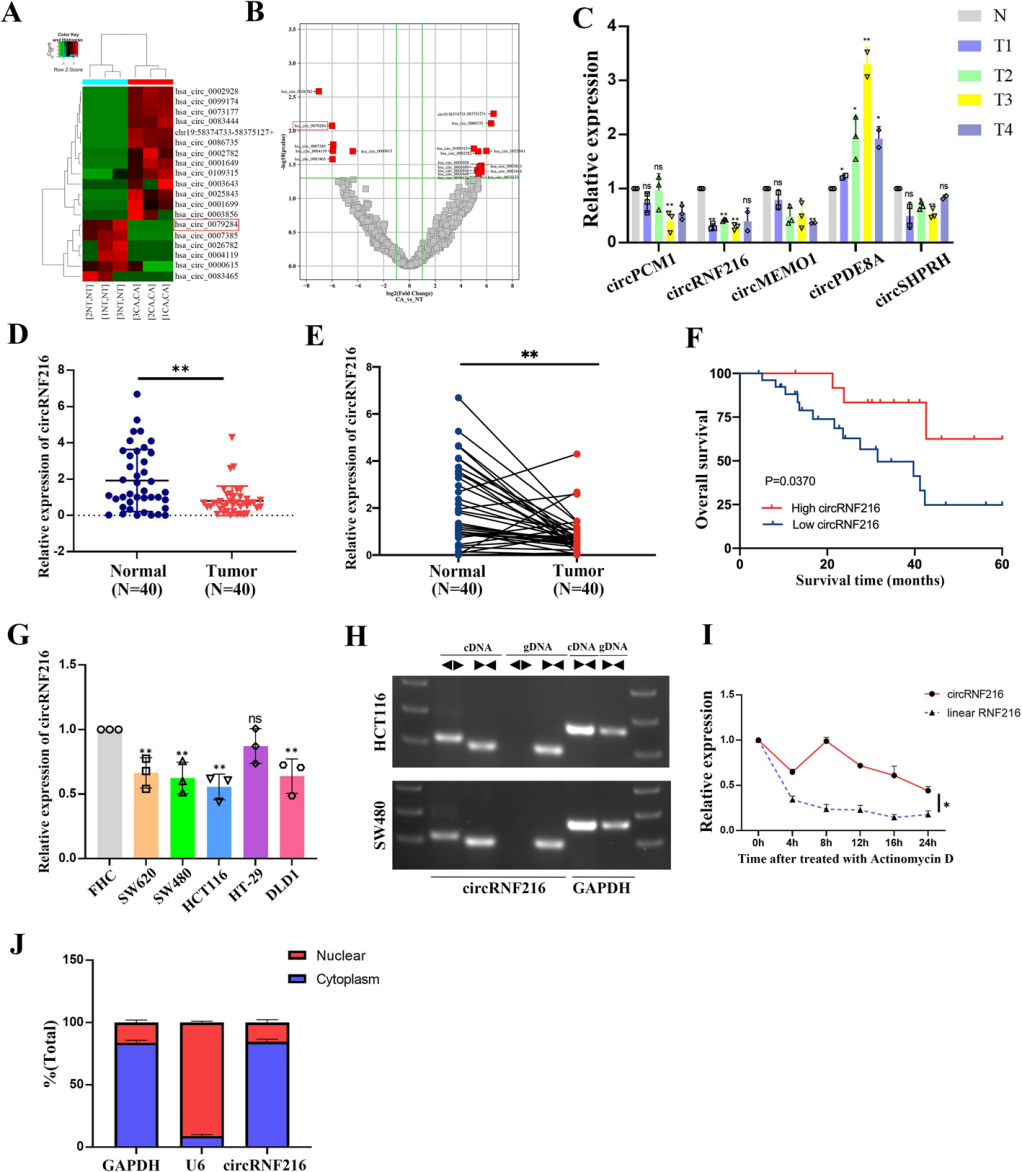
Identification and characterization of circRNF216 in CRC tissues. **A**, **B** Heatmap and Volcano plots generated from RNA-seq analysis shows differential expression of circRNAs. **C** qRT-PCR was performed on total RNA extracted from 4 pairs of CRC and normal tissues. **D**, **E** circRNF216 expression in 40 paired CRC and corresponding adjacent non-tumorous tissue samples was detected by qRT-PCR. **F** Survival curve was plotted by using Kaplan–Meier estimates. **G** qRT-PCR analysis of the relative circRNF216 levels in CRC cell lines. **H** The divergent primers detected circRNF216 in cDNA but not in gDNA. GAPDH was used as a positive control. **I** HCT116 cells were treated with actinomycin D to determine the half-life of circRNF216 and its linear transcript. **J** qRT-PCR was carried out to test nuclear and cytoplasmic RNA collected from equal numbers of CRC cells under each indicated condition. Data are represented by mean ± SD, *n* = 3, ns denotes not significant. *P < 0.05; **P < 0.01

To identify possible circRNAs candidates capable of regulating CRC development, ribosomal RNA (rRNA) was removed from total RNA extracted from 3 pairs of CRC tissues and corresponding adjacent non-tumor tissues for RNA sequencing. Consequently, 19 differentially expressed circRNAs were identified based on the thresholds of fold change (FC) (|log2FC|> 4) and *P*-value < 0.05 (Fig. 1A, B). Among them, 13 circRNAs were significantly up-regulated, whereas 6 were down-regulated. We selected and validated five circRNAs using qRT-PCR in four additional pairs of CRC tissues and adjacent normal tissues. According to the differential expression analysis, Hsa_circ_0079284 (circRNF216) was the most significantly down-regulated circRNA (Fig. 1C). To further validate whether circRNF216 expression in CRC tissues was consistent with RNA-seq data, qRT-PCR was employed to examine the RNA level of circRNF216. Results indicated that circRNF216 was down-regulated in 40 CRC tissue samples compared with adjacent normal samples (Fig. 1D, E). Subsequently, overall survival (OS) was calculated by Kaplan–Meier survival analysis. Consequently, CRC patients with low expression of circRNF216 exhibited lower OS rates compared to CRC patients with high levels of circRNF216 (Fig. 1F). Thus, the data demonstrated that circRNF216 might play a significant role in the CRC process.

### Expression and characterization of circRNF216 in CRC

Next, the circRNF216 expression levels in CRC cell lines (SW620, SW480, HCT116, HT-29, DLD1) were determined by qRT-PCR (Fig. 1G). Notably, HCT116 and SW480 cells demonstrated lower levels of circRNF216 expression among the tested cell lines. These two cell lines were then used to investigate the downstream regulatory pathways. To identify the circular structure of circRNF216, cDNA and gDNA were extracted from HCT116 and SW480 cells and used as the templates to amplify RNF216 mRNA and circRNF216 with divergent primers and convergent primers. Results indicated that divergent primers amplified circRNF216 in cDNA but not in gDNA (Fig. 1H). In addition, the stability of circRNF216 was determined by qRT-PCR after treatment with actinomycin D. It was observed that circRNF216 was more stable than linear RNF216 (Fig. 1I). Furthermore, we assessed the relative distribution of circRNF216 in the nucleus and cytoplasm of CRC cells by qRT-PCR analysis. It was showed that circRNF216 was predominantly cytoplasmic (Fig. 1J). In summary, circRNF216 has been identified as a circular RNA with significant implications in CRC. Its role and mechanisms warrant further investigation and exploration.

### CircRNF216 suppresses CRC cell migration and invasion in vitro

**Fig.2:**
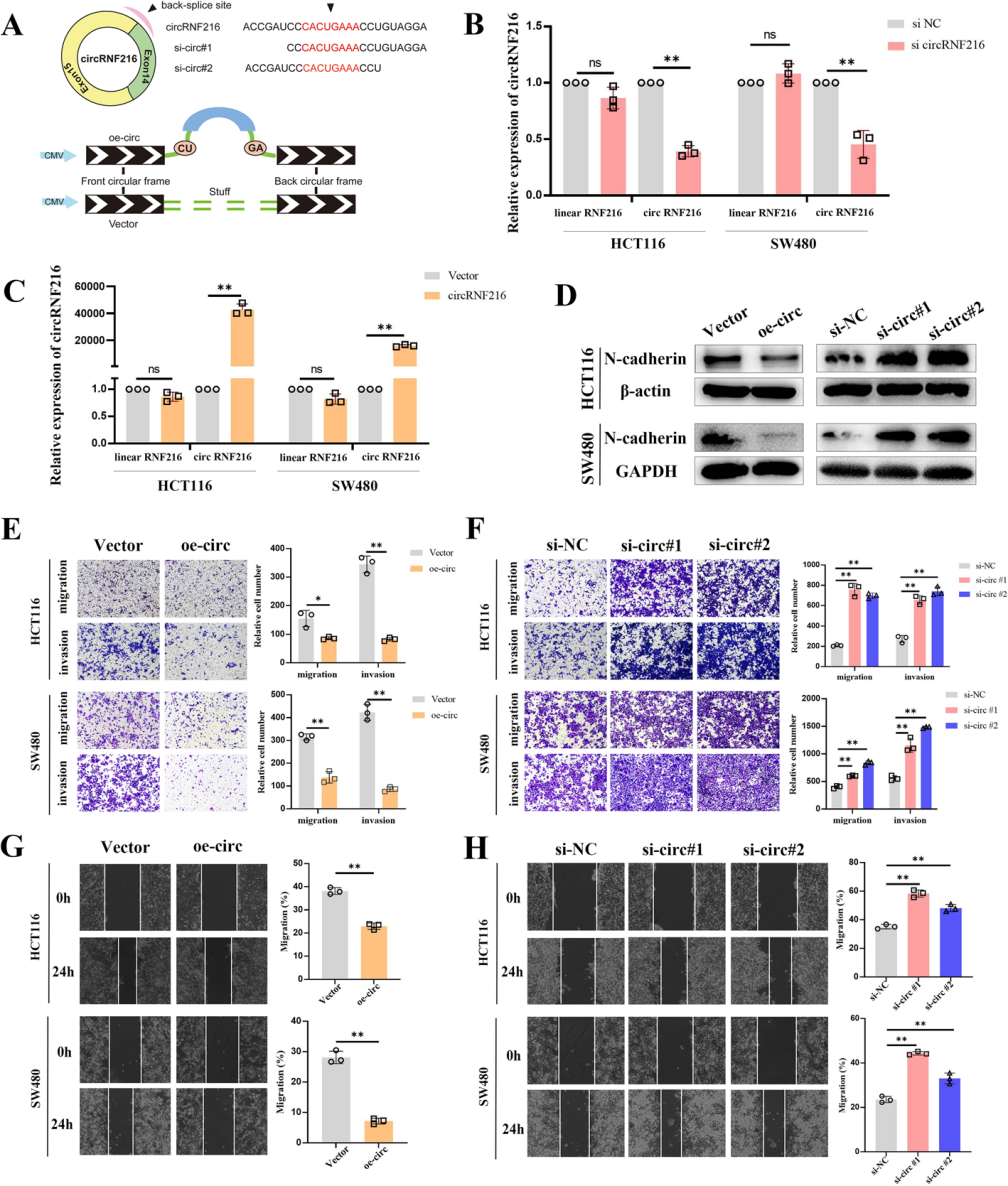
CircRNF216 suppresses migration and invasion of CRC cells. **A** Schematic diagram showing siRNA against circRNF216 and the overexpression vector selectively targeting the backsplice site sequences. **B** qRT-PCR analysis was performed to evaluate the expression levels of circRNF216 and linear RNF216 after silencing with the combined siRNA pool. **C** The expression of circRNF216 and linear RNF216 was analyzed by qRT-PCR after circRNF216 overexpression in CRC cells. **D** After transfection, WB assay was conducted to evaluate N-cadherin gene expression in HCT116 and SW480 cells. **E–H** Transwell and wound healing assays suggested that circRNF216 suppressed cell migration and invasion. Data are represented by mean ± SD, *n* = 3, ns denotes not significant. *P < 0.05; **P < 0.01

To explore whether circRNF216 is functionally linked to the CRC cell phenotype, two siRNA oligos against circRNF216 were designed at the splicing junction, which specifically targets circRNF216 but not linear RNF216 (Fig. 2A, B). In addition, we overexpressed circRNF216 in HCT116 and SW480 cells using the expression vector. Overexpression of circRNF216 was confirmed by qRT-PCR analysis, while transfection did not affect the linear RNF216 (Fig. 2C).

Notably, numerous studies suggested that EMT confers cancer cells with migratory and invasive ability [23]. Next, we investigated whether the expression of EMT-related gene expression was regulated by circRNF216 by western blot assays. The findings suggested several changes that occur in N-cadherin expression after transient transfection of circRNF216 (Fig. 2D, Additional file 1: Fig. S1). Transwell assays and wound healing assays results revealed circRNF216 could significantly decrease the migratory and invasive ability of HCT116 and SW480 cells (Fig. 2E, G), while circRNF216 knockdown promoted both in CRC cells (Fig. 2F, H). Taken together, these data provide evidence that circRNF216 inhibits N-cadherin expression to attenuate CRC cell migration and invasion.

### CircRNF216 functions as a sponge for miR-576-5p

**Fig. 3:**
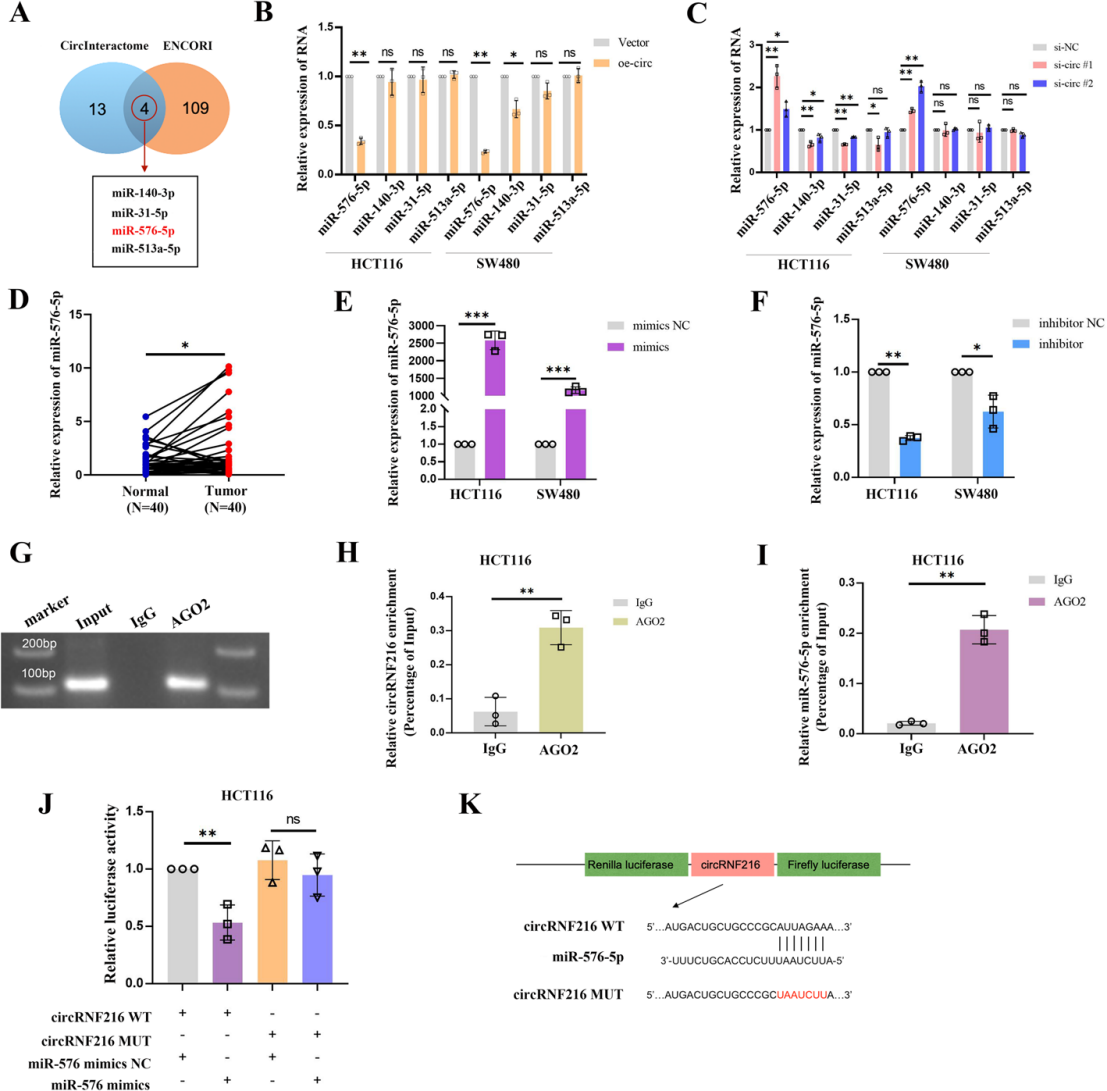
CircRNF216 acts as a sponge for miR-576-5p. **A** The target miRNAs within circRNF216 were predicted using online bioinformatic prediction tools CircInteractome and ENCORI. **B, C** After transfection, qRT-PCR was conducted to validate miRNAs expression in the HCT1116 and SW480 cell lines. **D** Relative miR-576-5p expression levels assessed by qRT-PCR in 40 paired CRC and corresponding normal tissue samples. **E, F** The RNA interference and overexpression efficiency were determined by qRT*-*PCR. **G-I** HCT116 cells were adopted to perform RIP assays using the AGO2 and IgG antibodies. **J** The relative luciferase activities were analyzed in HCT116 cells co-transfected with miR-576-5p mimics or miR-NC and luciferase reporter vectors circRNF216-WT or circRNF216-MUT. **K** Schematic diagram showing circRNF216-WT and circRNF216-MUT luciferase reporter vectors. Data are represented by mean ± SD, *n* = 3, ns denotes not significant. *P < 0.05; **P < 0.01

As sponging miRNAs is the most classical functional model of circRNA, we further explored whether circRNF216 could bind to one or more miRNAs to mediate CRC metastasis. Overlap of two prediction tools results demonstrated that four miRNAs were predicted to be more likely to bind to circRNF216 (Fig. 3A). Subsequently, we observed the changes in miRNA expression transfected circRNF216 in CRC cells, in comparison with the control group, and the results suggested that miR-576-5p had the most pronounced change in expression compared to the other candidates (Fig. 3B, C). Analysis of the 40 pairs of tissues mentioned above showed that miR-576-5p was significantly upregulated in CRC tissues (Fig. 3D). The effect of mimics and inhibitor of miR-576-5p was also verified (Fig. 3E, F). From well recognized, miRNAs are mainly bound with AGO2 proteins to form RNA-induced silencing complex (RISC) complexes [24], and subsequent RIP assays with AGO2 antibody showed that AGO2 antibody significantly enriched circRNF216 and miR-576-5p compared to the input group (Fig. 3G–I). The dual-luciferase reporter assay was used to further investigate the targeting interaction between circRNF216 and miR-576-5p, cotransfection of miR-576-5p mimics together with the circRNF216-WT caused significant repression of the luciferase reporter activity compared to NC mimics. In contrast, the luciferase activity of circRNF216-MUT was not affected (Fig. 3J, K). The above results suggest that circRNF216 can directly bind to miR-576-5p.

### MiR-576-5p promotes CRC cell migration and invasion in vitro by targeting ZC3H12C

**Fig. 4:**
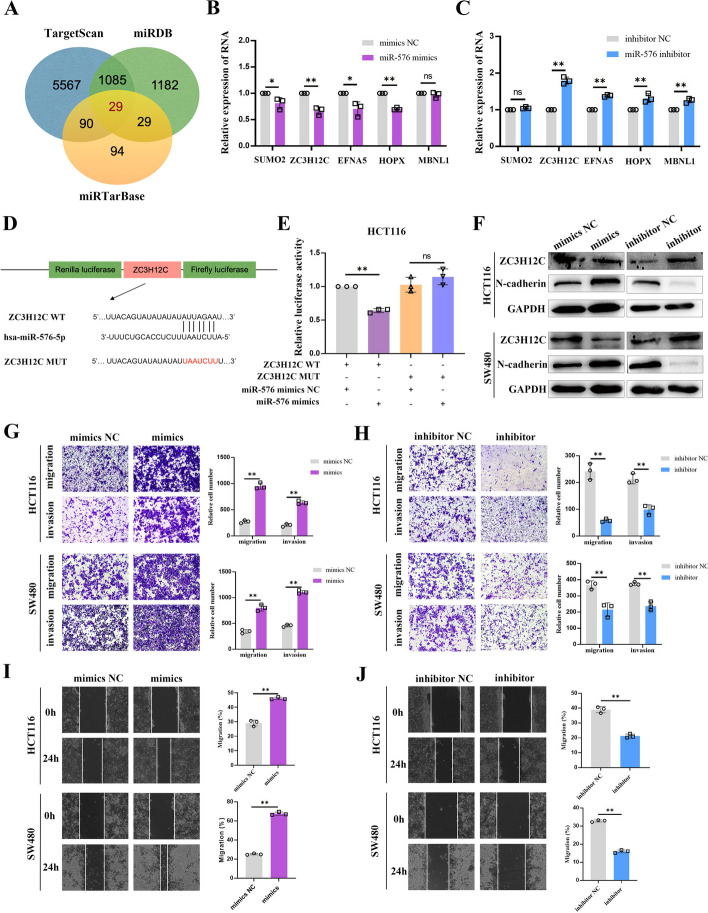
MiR-576-5p promotes CRC cell migration and invasion in vitro by targeting ZC3H12C. **A** Analysis of TargetScan, miRTarBase and miRDB databases predicted the genes as a target of miR-576-5p. **B, C** qRT-PCR analysis of mRNA expression of miR-576-5p-related genes. **D** Schematic diagram showing ZC3H12C-WT and ZC3H12C-MUT luciferase reporter vectors. **E** Luciferase activity in HCT116 cells co-transfected with ZC3H12C-WT or ZC3H12C-MUT and miR-576-5p mimics or NC, as determined using the dual-luciferase reporter assay system. **F** WB assay confirmed the ZC3H12C and N-cadherin protein expression in CRC cells after transfection with miR-576-5p. **G–J** The effect of miR-576-5p up-regulation or down-regulation on cell migratory capability was evaluated by transwell and wound healing assays in HCT116 and SW480 cells. Data are represented by mean ± SD, *n* = 3, ns denotes not significant. *P < 0.05; **P < 0.01

Following this lead, TargetScan, miRDB, and miRTarBase databases were used to identify the target genes for miR-576-5p (Fig. 4A). After reviewing the relevant literature, we screened five from 29 candidate genes for validation. Next, target genes expression in the miR-576-5p mimics and inhibitor groups were assessed using qRT-PCR, and ZC3H12C was selected for subsequent studies due to its more stable and significant changes (Fig. 4B, C).

Furthermore, to confirm the bioinformatics predictions, ZC3H12C sequences containing the wild-type (WT) or mutated (MUT) putative miR-576-5p binding sites were cloned into the pmirGLO vector, and then the miR-576-5p was overexpressed in cells using mimics. Experimental results indicated that the ZC3H12C 3'UTR-WT sequence carrying the luciferase reporter vector was significantly reduced luciferase activity in the miR-576-5p mimics group, however, there was no significant difference in the ZC3H12C-Mut (Fig. 4D, E). To assess the clinical features of ZC3H12C expression, we used the TNMplot and TIMER website to examine the expression of ZC3H12C in various tumor tissues and normal tissues. The results demonstrated that ZC3H12C expression was significantly lower in colon adenocarcinoma (COAD) and rectal adenocarcinoma (READ) than normal tissues (Additional file 1: Fig. S2A, B). Next, we evaluated the immunohistochemical staining of ZC3H12C via HPA database. Results showed that ZC3H12C was weakly marked in colorectal cancer and mainly located in cytoplasm (Additional file 1: Fig. S3A, B). A protein–protein interaction (PPI) network was constructed using the BioGRID website to analyze ZC3H12C protein interactions (Additional file 1: Fig. S4A). In addition, to investigate the biological role of ZC3H12C in CRC, volcano plots and heatmaps were generated to visualize the differential expression of genes associated with ZC3H12C in CRC through the Linkedomics database (Additional file 1: Fig. S4B, C). KEGG pathway analysis indicated that ZC3H12C was involved in cell cycle and DNA replication pathways that are associated with tumor progression (Additional file 1: Fig. S4D).

We further evaluated the biological functions of miR-576-5p in CRC cells. miR-576-5p overexpression diminished the ZC3H12C expression level and increased the expression of N-cadherin, while miR-576-5p inhibitors showed the reverse results (Fig. 4F). The metastatic abilities of HCT116 and SW480 were greatly boosted by increasing miR-576-5p expression (Fig. 4G, I) and significantly diminished with decreasing miR-576-5p expression (Fig. 4H, J) according to wound healing and transwell assay data. These results confirmed the promoting effect of miR-576-5p on migration and invasion in CRC cells.

### CircRNF216 suppresses CRC cell migration and invasion though circRNF216/miR-576-5p/ZC3H12C axis

**Fig. 5:**
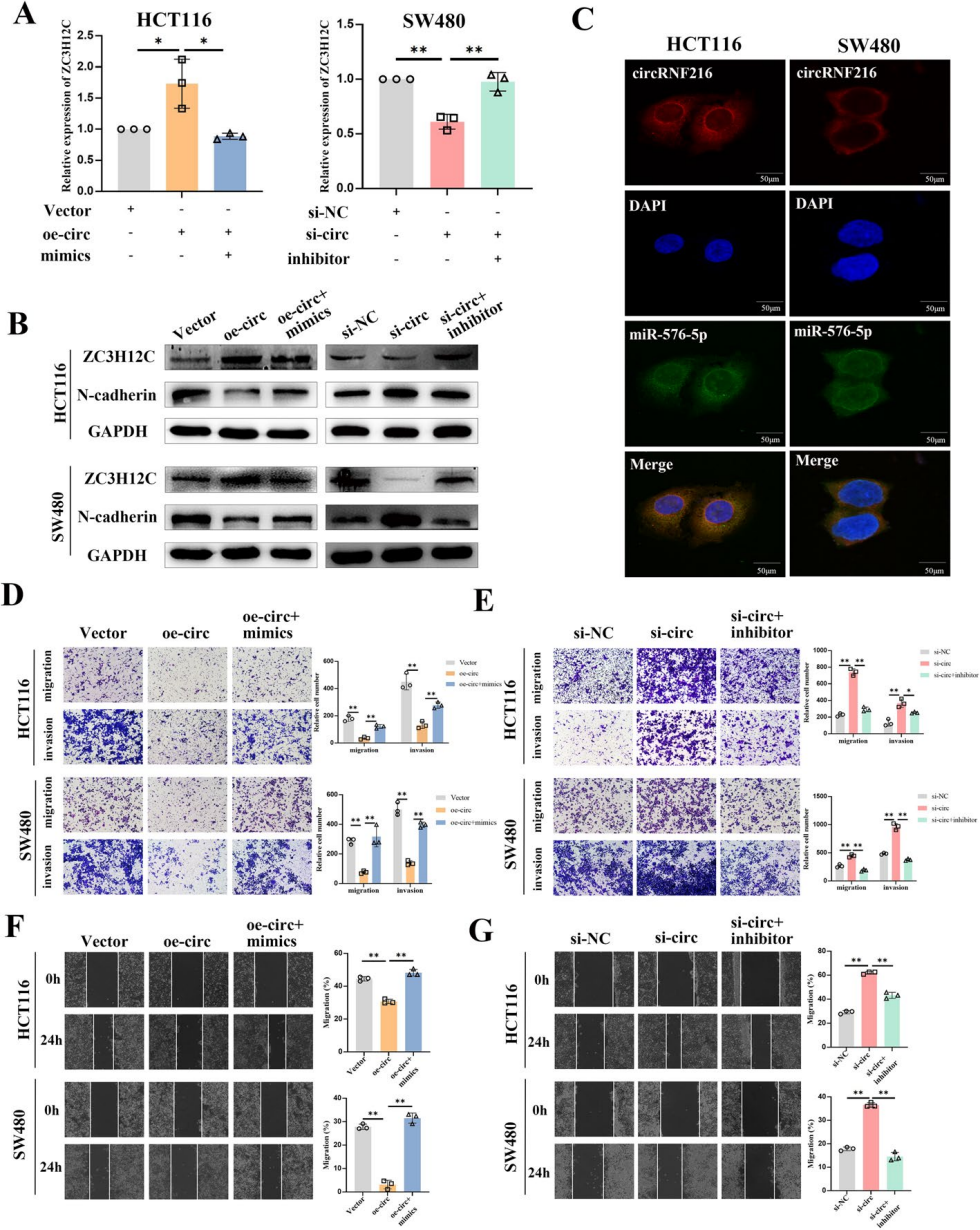
CircRNF216 suppresses CRC cell migration and invasion by relieving miR-576-5p repression of ZC3H12C expression. **A** qRT-PCR showed that miR-576-5p reversed the circRNF216-induced enhancement of ZC3H12C expression.** B** Expression of ZC3H12C and N-cadherin in CRC cells was analyzed by WB assay, with GAPDH as a control. **C** FISH analysis of the co-localization of circRNF216 (red) and miR-576-5p (green) in HCT116 and SW480 cells. **D–G** Motility ability of CRC cells co-transfected with oe-circRNF216 and miR-576-5p mimics or si-circRNF216 and miR-576-5p inhibitor was assessed by wound healing and transwell assays. Data are represented by mean ± SD, *n* = 3, ns denotes not significant. *P < 0.05; **P < 0.01

Rescue experiments using miR-576-5p mimics and inhibitors were performed to explore whether circRNF216 exerted the tumor-suppressive effects via the circRNF216/miR-576-5p/ZC3H12C axis. MiR-576-5p mimics obviously abrogated the ZC3H12C expression induced by circRNF216 overexpression, as evidenced by the increased N-cadherin expression from qRT-PCR and WB assays. Meanwhile, miR-576-5p inhibitor reversed the regulatory effects caused by silencing circRNF216 (Fig. 5A, B). Furthermore, given that the ceRNA machinery of circRNA usually occurs in the cytoplasm, FISH results indicated that circRNF216 and miR-576-5p were co-localized in the cytoplasm (Fig. 5C). Meanwhile, we sought to determine whether the biological function of circRNF216 in CRC cells could also be reversed by miR-576-5p mimics or inhibitor. The findings showed that miR-576-5p mimics counteracted the inhibitory effect induced upon overexpression of circRNF216 (Fig. 5D, F), while miR-576-5p knockdown significantly offset the facilitative effect of circRNF216 downregulation on the metastasis capacity of CRC cells (Fig. 5E, G). In conclusion, these data suggested that circRNF216 could function as a ceRNA by sponging miR-576-5p to regulate ZC3H12C expression and inhibit CRC cell metastasis.

### CircRNF216 is correlated with CD8^+^ T cell infiltration via regulating ZC3H12C

**Fig. 6:**
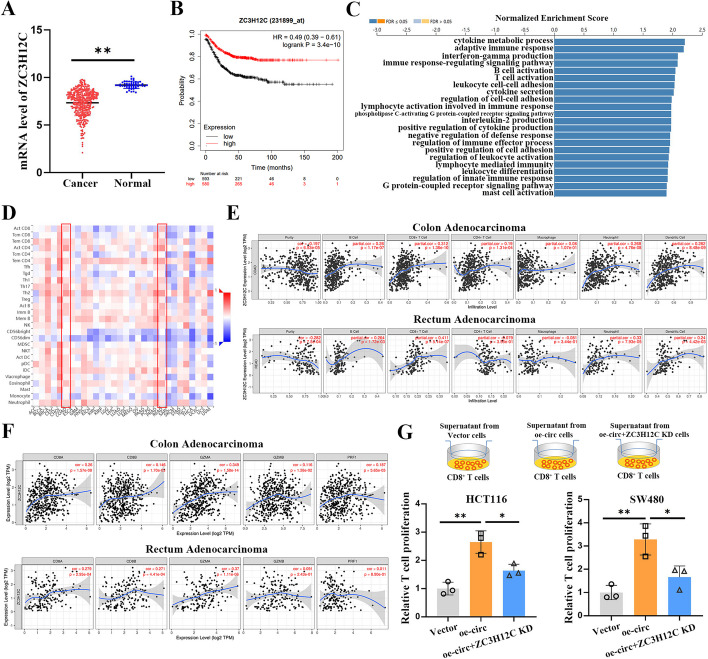
CircRNF216 is correlated with CD8^+^ T cell infiltration via regulating ZC3H12C. **A** TCGA database was utilized to examine ZC3H12C expression in CRC tissues and normal tissues. **B** Low ZC3H12C expression was related with a poor overall survival (OS) according to the Kaplan–Meier Plot database. **C** GO analysis of ZC3H12C in CRC. **D** The correlations of immune cell infiltrations and ZC3H12C were accessed by TISIDB database. In the analysis, positive correlations were represented by the color red, while negative correlations were represented by the color blue. The intensity of the color reflected the strength of the correlation, with darker shades indicating stronger associations. **E** TIMER web server was utilyzed to assess the correlation of ZC3H12C and immune cells in colon adenocarcinoma and rectum adenocarcinoma. **F** The correlations between ZC3H12C expression and CD8A, CD8B, GZMA, GZMB, PRF1 in colon adenocarcinoma and rectum adenocarcinoma were analyzed by TIMER web server. **G** CircRNF216 promoted the proliferation of CD8^+^ T cells, while ZC3H12C deficiency reduced the proliferation CD8^+^ T cells in HCT116 and SW480 cells. Data are represented by mean ± SD, *n* = 3, ns denotes not significant. *, *P* < 0.05; **, *P* < 0.01

Bioinformatics analysis showed that ZC3H12C expression was significantly lower in CRC tissues than normal tissues (Fig. 6A). Furthermore, low ZC3H12C expression was related with a poor overall survival (OS) according to the Kaplan–Meier Plot database (Fig. 6B). GO (Fig. 6C) and GESA analysis (Additional file 1: Fig. S5) revealed that ZC3H12C was involved in immune response in CRC, including adaptive immune response, interferon-gamma production, immune response-regulating signaling pathway, B cell activation, T cell activation. Therefore, we further investigated the associations between immune cell infiltrations and ZC3H12C in multiple cancer types using TISIDB (Fig. 6D). The analysis revealed a positive correlation between ZC3H12C expression and the presence of CD8 + T cells. This indicates a potential role of ZC3H12C in modulating the tumor immune microenvironment by influencing CD8 + T cell infiltration. TIMER web server was enrolled to access the correlation of ZC3H12C and immune cells in colon adenocarcinoma and rectum adenocarcinoma. Results showed that ZC3H12C was positively correlated with CD8^+^ T cells infiltration (Fig. 6E). In addition, the positive correlations between ZC3H12C expression and highly connected CD8^+^ T genes were also visualized, including CD8A, CD8B, GZMA, GZMB, and PRF1 in colon adenocarcinoma and rectum adenocarcinoma (Fig. 6F). Furthermore, circRNF216 also promoted the proliferation of CD8^+^ T cells, and ZC3H12C deficiency reduced the proliferation CD8^+^ T cells in HCT116 and SW480 cells (Fig. 6G). These data indicated that circRNF216 may promote CD8^+^ T cells infiltration probably by upregulating ZC3H12C.

### CircRNF216 inhibited the tumor metastasis and recruited CD8^+^ T cells via ZC3H12C in vivo

**Fig. 7:**
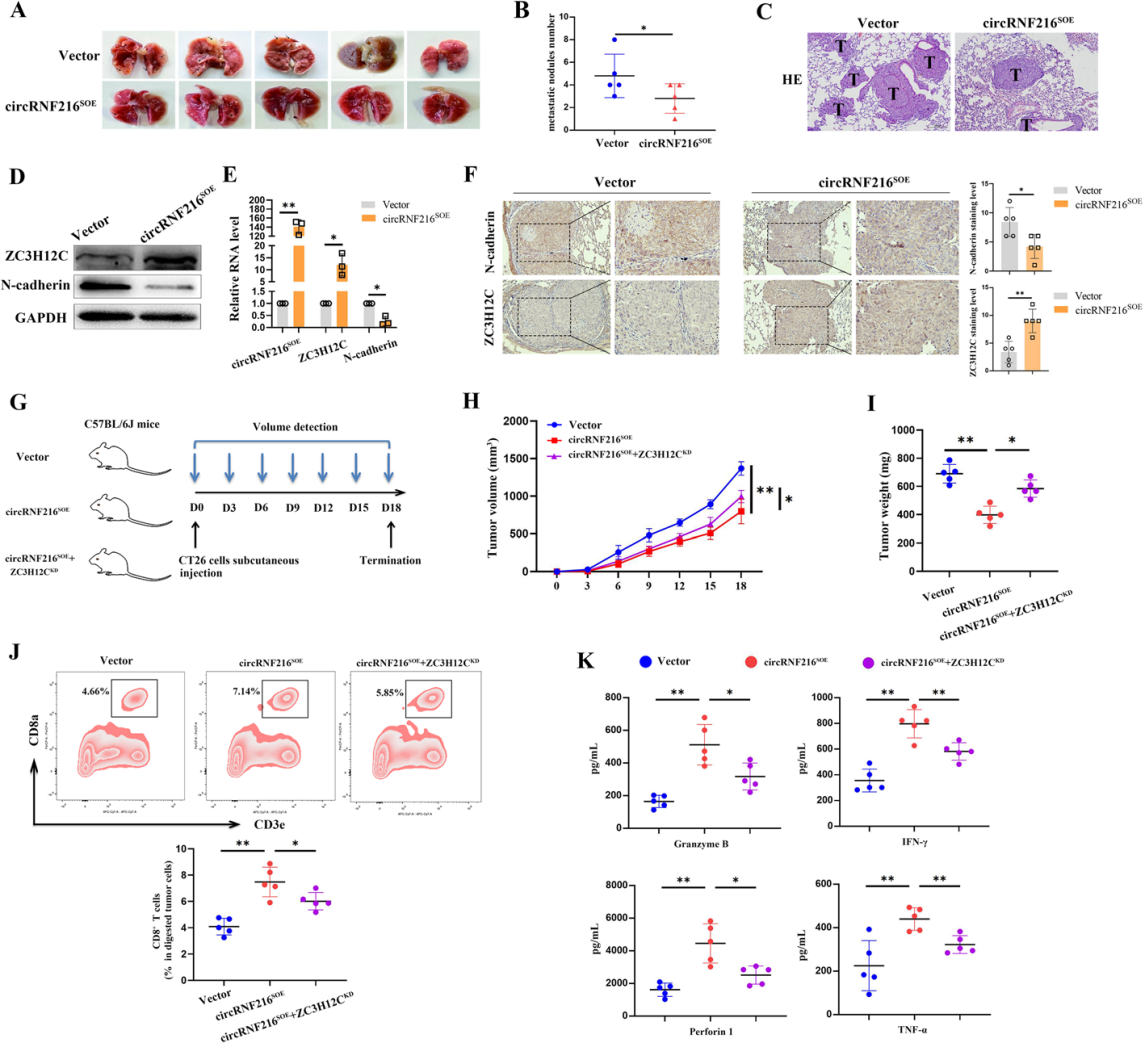
CircRNF216 inhibited the tumor metastasis and recruited CD8^+^ T cells via ZC3H12C in vivo. **A, B** Images and metastatic nodules number of the nude mice lungs following injections of Vector and circRNF216^soe^ in HCT116 cells. **C** H&E staining indicated less metastasis after circRNF216 overexpression, with arrows indicating the metastatic foci. **D, E** ZC3H12C and N-cadherin expression levels were observed after lentiviral transfection in HCT116 cells by WB and q-PCR assays. **F** Representative IHC images showing ZC3H12C and N-cadherin expression in metastatic nodules. **G** Schematic of the syngeneic CRC model. **H, I** Tumor volumes and tumor weight of mice in subcutaneous CRC model. **J** Flow cytometry analysis showed that circRNF216 overexpression increased the number of tumor-infiltrating CD8^+^ T cells, while ZC3H12C knockdown decreased tumor infiltrating CD8^+^ T cells in C57BL/6J mice. **K** circRNF216 overexpression tumors had significantly higher amounts of Granzyme B, IFN-γ, TNF-α and perforin1 than Control tumors, while knockdown of ZC3H12C rescued circRNF216 overexpression induced high soluble factors in C57BL/6J mice. Data are represented by mean ± SD, *n* = 3, ns denotes not significant. *P < 0.05; **P < 0.01

To further investigate whether circRNF216 affected the metastasis of CRC in vivo, HCT116 cells stably expressing the control vector or circRNF216 were injected into the tail vein of female BALB/C nude mice. After six weeks, the lungs of control and experimental mice were observed for metastasis. Upon gross inspection, nude mice in circRNF216 overexpression group had less metastatic tumors in their lungs (Fig. 7A, B). H&E staining showed significantly reduced tumor formation in the circRNF216 overexpression group (Fig. 7C). These data suggest that circRNF216 inhibits cancer metastasis in vivo. In addition, WB and q-PCR results indicated that ZC3H12C expression was upregulated in the circRNF216 overexpression group, while N-cadherin was downregulated (Fig. 7D, E). Results from IHC also showed that concomitant with the loss of N-cadherin, the upregulation of ZC3H12C expression is observed, which is consistent with our in vitro results (Fig. 7F). Next we aimed to investigate the effect of circRNF216 on tumor immune cells infiltration in mouse models. A syngeneic subcutaneous CRC model was established by mouse CRC cell line CT26 in C57BL/6J mice (Fig. 7G). q-PCR results showed that the expression of ZC3H12C was upregulated in the circRNF216 overexpression group of CT26 cells, while miR576-5p was downregulated (Additional file 1: Fig. S6). Both tumor volumes and tumor weights were significantly diminished after circRNF216 overexpression, while ZC3H12C knockdown enhanced the tumor volumes and tumor weights (Fig. 7GH, I). To explore the immune cells infiltration in tumor tissues, we conducted flow cytometry analysis and found that circRNF216 overexpression enhanced the number of tumor-infiltrating CD8^+^ T cells, while ZC3H12C knockdown decreased tumor infiltrating CD8^+^ T cells in C57BL6 mice (Fig. 7J). Furthermore, circRNF216 overexpression tumors had significantly higher amounts of Granzyme B, IFN-γ, TNF-α and perforin1 than Control tumors, while knockdown of ZC3H12C rescued circRNF216 overexpression induced high soluble factors in C57BL6 mice (Fig. 7K). In conclusion, our data provide in vivo evidence documenting the relationship between circRNF216 and CRC progression.

## Discussion

CircRNAs are RNA molecules that have a closed continuous loop structure [25]. CircRNAs are more resistant to degradation by ribonuclease R (RNase R) compared to linear RNAs due to their covalently closed-loop structure and absence of free terminal ends, which contributes to the enhanced stability of circRNAs [26]. Thousands of circRNAs have been identified in eukaryotes thanks to the advances in high-throughput sequencing and unbiased computational approaches [27]. Furthermore, circRNAs have been implicated in tissue and organ development, along with processes such as neurodegeneration, immunity, and tumor formation [28, 29]. However, the function of circRNAs in CRC progression and anti-tumor immunity is mainly unknown and warrants additional investigation.

Through utilizing a combined analysis of genomics, biochemistry and cell biology, we identified a circular RNA circRNF216, down-regulated in CRC and linked to overall survival of CRC patients. circRNF216 is a 321 bp exonic circRNA, contains two exons of its parent gene RNF216. In mechanistic experiments, overexpression of circRNF216 was found to upregulate ZC3H12C by specifically sponging miR-576-5p. ZC3H12C, which harbors a CCCH zinc finger domain and an NYN RNase domain, and the physiological function of the RNase depends on its degradation target [30, 31]. Results suggested that ZC3H12C inhibited CRC metastasis by downregulating N-cadherin. Furthermore, previous study concluded that the mRNA degradation activity of ZC3H12C is non-specific and whether additional binding partners are required to enhance the specificity of ZC3H12C for its degradation targets also deserves further investigation [32]. Recently, circRNA has gained considerable attention as a component of ribonucleoprotein or RNA-RNA complexes, which are involved in a variety of events that affect cellular function, physiology, and disease states [33–35]. As far as the current studies are concerned, the use of N-cadherin antagonists as oncological therapeutic agents in the clinical setting is still at an early stage of the investigation. Only a few clinical trials have been performed with ADH-1 and no trials with other N-cadherin antagonists have been performed [36, 37]. Consequently, targeting the inhibition of N-cadherin before its translation into a mature proprotein could be considered as a viable alternative pathway to inhibit metastasis. In summary, circRNF216 appears to play a role in conferring resistance to tumor metastasis through its interaction with miR-576-5p. Moreover, in vivo animal studies have provided further confirmation of the anti-oncogenic properties of circRNF216.

**Fig. 8:**
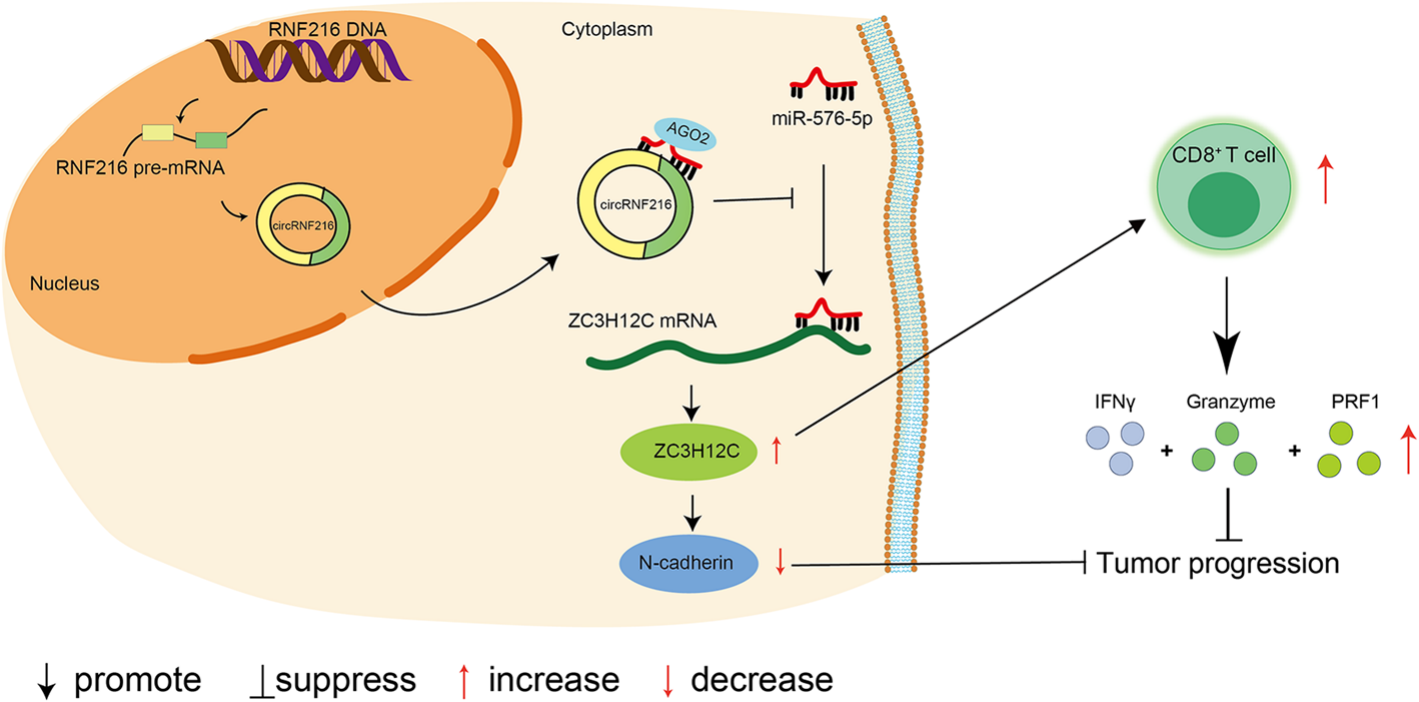
Hypothetical model illustrating the roles of circRNF216 in the inhibition of tumor progression in CRC. CircRNF216 acts as a sponge for miR-576-5p, alleviating miR-576-5p repression on its target ZC3H12C, which in turn downregulated N-cadherin. In addition, circRNF216 could augment the recruitment of CD8^+^ T cells via ZC3H12C, thereby triggering the release of soluble factor, ultimately inhibiting the progression of CRC

Immunotherapies are the most rapidly growing drug class and have changed the landscape of cancer treatment by triggering long-term antitumor immunity [38]. Emerging evidences indicate that responses to immunotherapy rely on tumor infiltration by CD8^+^ T cells, CD4^+^ T cells, DC cells that are able to recognize and eradicate tumor cells [39]. Of note, intratumoral CD8^+^ T cells in TIME are a critical positive predictor of clinical outcomes [40]. Previous studies showed that circRNA inhibited tumors development via acting as vaccine adjuvant to induce antigen-specific T cell activation [41]. A subset of circRNAs with double-stranded structure could inhibit innate immune responses [42]. CircRNAs leads to innate immunity gene expression that presents protection against viral infection [43]. These data signify the function of circRNA in triggering cellular immune response, whereas manners and mechanism of anti-tumor immune responses induced by circRNAs in CRC progression remains to be elucidated. Here, we exhibited circRNF216 triggered immune defense response in CRC models. Based on the in vitro and in vivo experiments in this study, we first report that circRNF2161 enhanced the recruitment of CD8^+^ T cells and inhibited CRC progression. Our findings broaden the understanding of mechanism in circRNAs activating anti-tumor immunity in CRC (Fig. 8).

## Conclusions

In summary, given that circRNF216 is a type of circular non-coding RNA with tumor suppressor properties, we investigated the molecular mechanism of circRNF216 in CRC. The experiments identified circRNF216 as a critical modulator of metastasis, as it could repress N-cadherin expression via adsorbing miR-576-5p to upregulate ZC3H12C expression. In addition, circRNF216 regulates the infiltration level of CD8^+^ T cells by upregulating ZC3H12C. However, it is important to note that there may be other crucially down-regulated circRNAs involved in ZC3H12C-induced CRC progression. Furthermore, the potential association of circRNF216 with other immune cells requires further confirmation. Finally, as understanding of the role of circRNAs in diverse developmental processes and disorders continues to expand, uncovering the molecular partners of circRNAs influencing cellular functions will be crucial for the development of effective interventions.

### Supplementary Information

**Additional file1: Figure S1. **Evaluation of the EMT markers following circRNF216 overexpression or knockdown.** Figure S2.** Expression of ZC3H12C in CRC.** A **Data derived from the TIMER database showed ZC3H12C was highly expressed in normal tissues compared with COAD and READ. **B **ZC3H12C was highly expressed in normal tissues compared with COAD according to TNMplot web tool. **Figure S3. **Analysis of ZC3H12C and clinical features of in CRC.** A-B **HPA database showed that ZC3H12C was weakly stained in colorectal cancer and mainly located in cytoplasm. **Figure S4. **Biological function of ZC3H12C in CRC.** A **Investigating notable protein interactions related to ZC3H12C in the BioGRID database. **B-C** Heatmaps and volcano plots were used to identify the genes exhibiting differential expression of ZC3H12C in CRC. **D** Performing KEGG pathway analysis on the differentially expressed genes involving ZC3H12C in CRC. **Figure S5. **GESA analysis of ZC3H12C in CRC.** Figure S6. **Detection of miR576-5p and ZC3H12C RNA level after circRNF216 overexpression in CT26 cells.** Table S1.** Primers used in this study.

## Data Availability

All data generated or analyzed in the current study are available from the corresponding author on reasonable request.

## Abbreviations

TIME: Tumor immune microenvironment
CRC: Colorectal cancer
circRNAs: Circular RNAs
RIP: RNA immunoprecipitation
FISH: Fluorescent in situ hybridization
ceRNA: Competitive endogenous RNA
MREs: MiRNA response elements
RBPs: RNA binding proteins
CTLs: CD8^+^ cytotoxic T lymphocytes
EMT: Epithelial-mesenchymal transition
RNA-seq: RNA sequencing
qRT-PCR: Quantitative real-time PCR
WB: Western-blot
H&E: Hematoxylin and eosin
IHC: Immunohistochemical
ELISA: Enzyme-linked immunosorbent assay
gDNA: Genomic DNA
OS: Overall survival
RNase R: Ribonuclease R
