# Supplementary material for: Regulation of tumor metastasis and CD8+ T cells infiltration by circRNF216/miR-576-5p/ZC3H12C axis in colorectal cancer
Source: Cell Mol Biol Lett. 2024 Jan 24;29:19. doi: 10.1186/s11658-024-00539-z (PMC10809481; doi:10.1186/s11658-024-00539-z)
Supplement: Supplementary file 1 — Additional file1: Figure S1. Evaluation of the EMT markers following circRNF216 overexpression or knockdown. Figure S2. Expression of ZC3H12C in CRC. A Data derived from the TIMER database showed ZC3H12C was highly expressed in normal tissues compared with COAD and READ. B ZC3H12C was highly expressed in normal tissues compared with COAD according to TNMplot web tool. Figure S3. Analysis of ZC3H12C and clinical features of in CRC. A-B HPA database showed that ZC3H12C was weakly stained in colorectal cancer and mainly located in cytoplasm. Figure S4. Biological function of ZC3H12C in CRC. A Investigating notable protein interactions related to ZC3H12C in the BioGRID database. B-C Heatmaps and volcano plots were used to identify the genes exhibiting differential expression of ZC3H12C in CRC. D Performing KEGG pathway analysis on the differentially expressed genes involving ZC3H12C in CRC. Figure S5. GESA analysis of ZC3H12C in CRC. Figure S6. Detection of miR576-5p and ZC3H12C RNA level after circRNF216 overexpression in CT26 cells. Table S1. Primers used in this study. [file 11658_2024_539_MOESM1_ESM.docx]

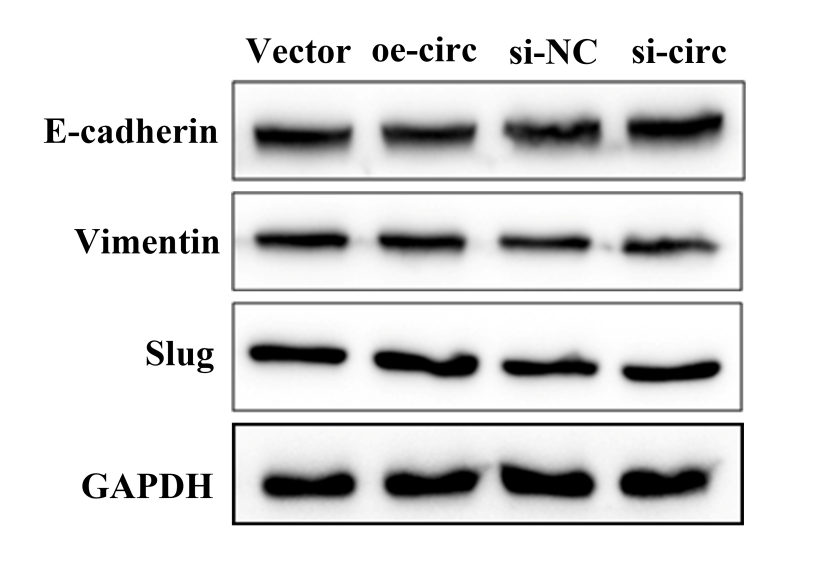


**Fig. S1 Evaluation of the EMT markers following circRNF216 overexpression or knockdown.**

**
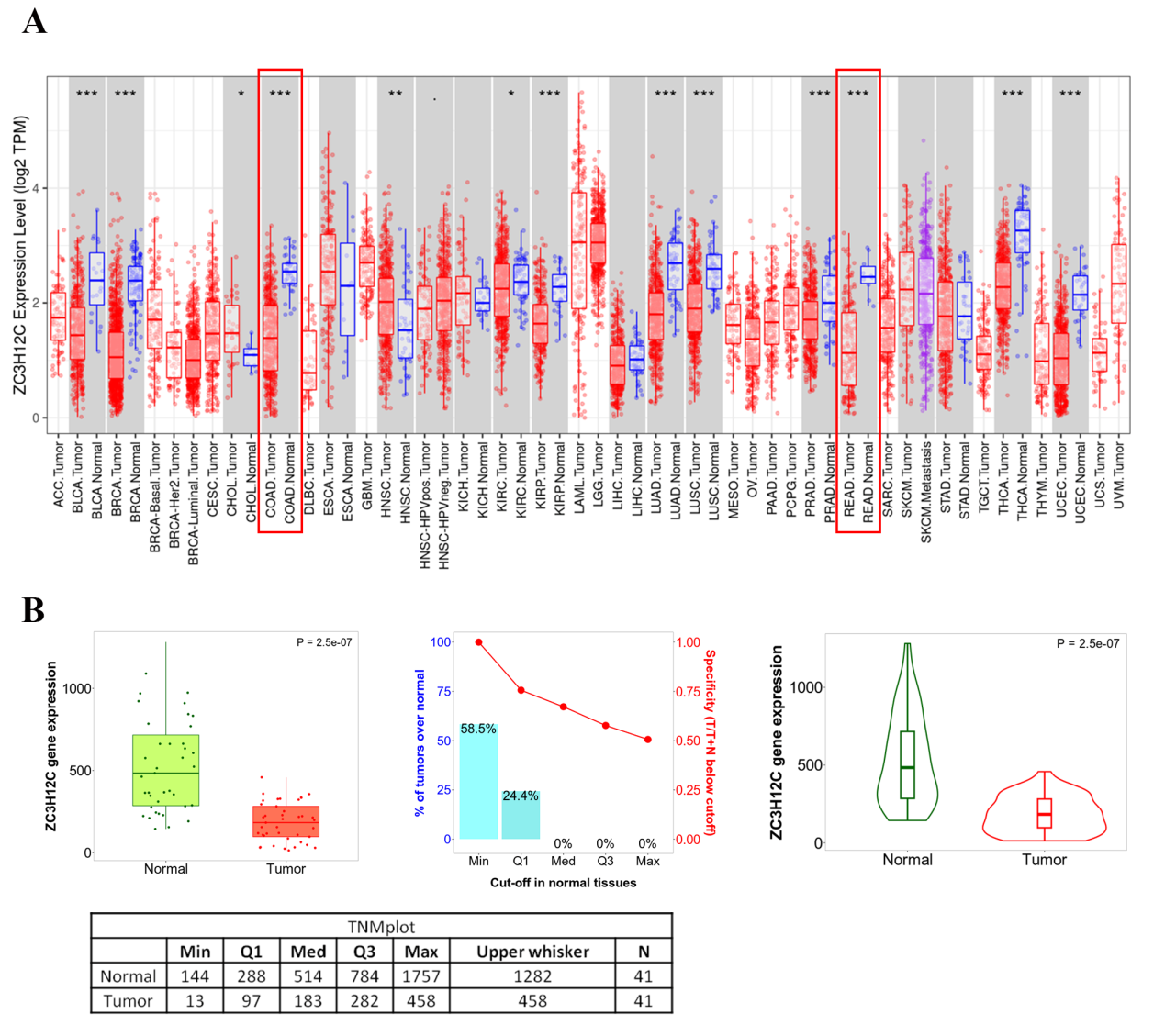
**

**Fig. S2** **Expression of ZC3H12C in CRC. A** Data derived from the TIMER database showed ZC3H12C was highly expressed in normal tissues compared with COAD and READ. **B** ZC3H12C was highly expressed in normal tissues compared with COAD according to TNMplot web tool.


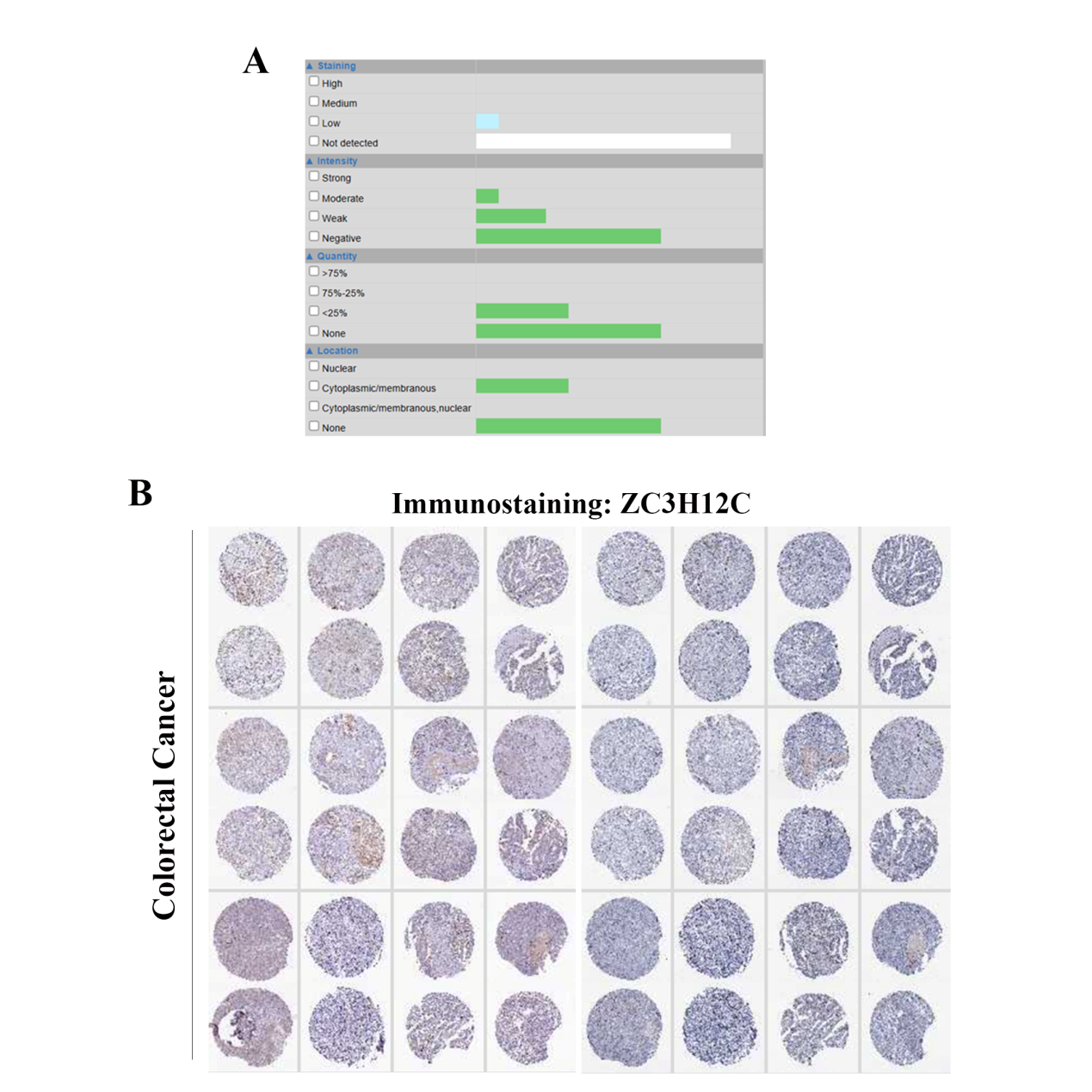


**Fig. S3 Analysis of ZC3H12C and clinical features of in CRC. A-B** HPA database showed that ZC3H12C was weakly stained in colorectal cancer and mainly located in cytoplasm.


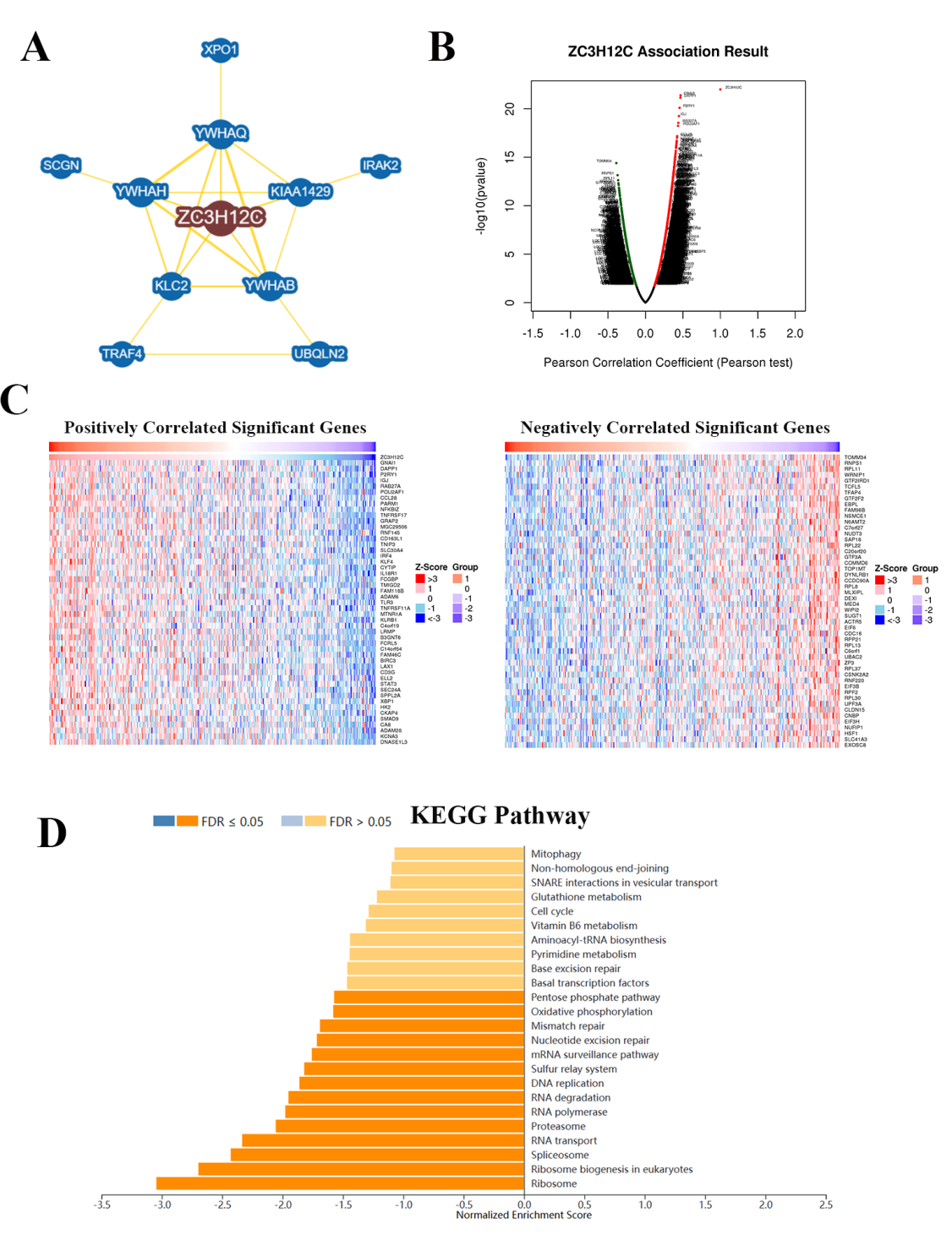


**Fig. S4 Biological function of ZC3H12C in CRC. A** Investigating notable protein interactions related to ZC3H12C in the BioGRID database. **B-C** Heatmaps and volcano plots were used to identify the genes exhibiting differential expression of ZC3H12C in CRC. **D** Performing KEGG pathway analysis on the differentially expressed genes involving ZC3H12C in CRC.


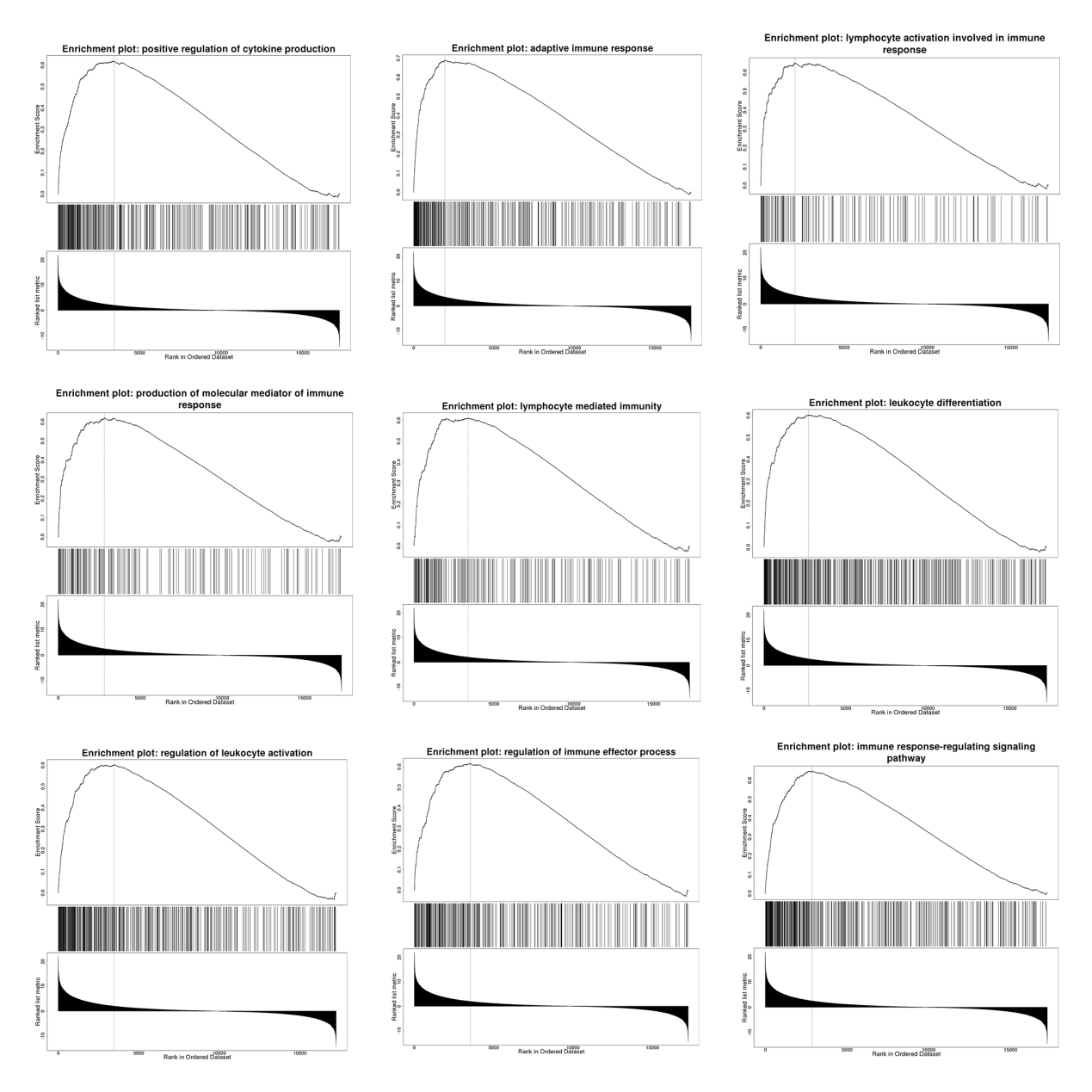


**Fig. S5 GESA analysis of ZC3H12C in CRC.**

**
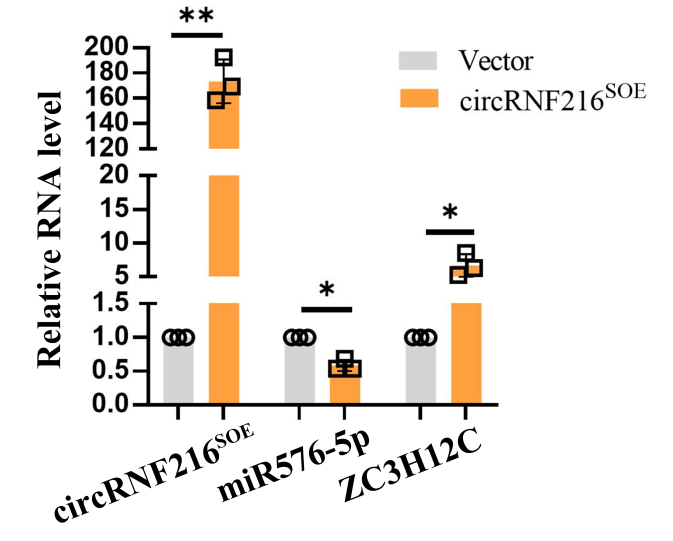
**

**Fig. S6 Detection of miR576-5p and ZC3H12C RNA level after circRNF216 overexpression in CT26 cells.**

| **Table S1** Primers used in this study | | |
| --- | --- | --- |
| Name | Primer | Sequence (5’-3’) |
| circPCM1 | Forward primer | TCCAGACTTCCCTCCAGGCT |
|  | Reverse primer | ACACCTCAGATGTTTGAGTCTGCT |
| circRNF216 | Forward primer | CCCCTTGCCAGGAGTGTTCA |
|  | Reverse primer | GCTCTTCACAGGTGAGGCCA |
| circMEMO1 | Forward primer | TGTTTGAACGCATGTCTCTGCA |
|  | Reverse primer | AAGCATGGGCAGCACAAGAC |
| circPDE8A | Forward primer | TGATCCCCATGCCAATGACCT |
|  | Reverse primer | GCTTCTGTCACAGGCATGGG |
| circSHPRH | Forward primer | AGGGTGCACCGAATTGGACA |
|  | Reverse primer | AGTTCTGACCACAGCTTCCACT |
| linear RNF216 | Forward primer | GACTGCTGCCCGCATTAGAAAATG |
|  | Reverse primer | CGGTCCAGAGAGAGCATCTTGAAC |
| 18S | Forward primer | CCTGAGAAACGGCTACCACAT |
|  | Reverse primer | CACCAGACTTGCCCTCCA |
| miR-140-3p | Stem-loop Primer | GTCGTATCCAGTGCAGGGTCCGAGGTATTCGCACTGGATACGACCCGTGG |
|  | Forward primer | GCTGCGTACCACAGGGTAGAA |
|  | Reverse primer | AGTGCAGGGTCCGAGGTATT |
| miR-31-5p | Stem-loop Primer | GTCGTATCCAGTGCAGGGTCCGAGGTATTCGCACTGGATACGACAGCTAT |
|  | Forward primer | GCGAGGCAAGATGCTGGC |
|  | Reverse primer | AGTGCAGGGTCCGAGGTATT |
| miR-576-5p | Stem-loop Primer | GTCGTATCCAGTGCAGGGTCCG AGGTATTCGCACTGGATACGAC |
|  | Forward primer | GCGCGATTCTAATTTCTCCAC |
|  | Reverse primer | AGTGCAGGGTCCGAGGTATT |
| miR-513a-5p | Stem-loop Primer | GTCGTATCCAGTGCAGGGTCCGAGGTATTCGCACTGGATACGACATGACA |
|  | Forward primer | GCTGCGTTCACAGGGAGG |
|  | Reverse primer | AGTGCAGGGTCCGAGGTATT |
| U6 | Forward primer | GCTTCGGCAGCACATATACTAAAAT |
|  | Reverse primer | CGCTTCACGAATTTGCGTGTCAT |
| SUMO2 | Forward primer | CCGACGAAAAGCCCAAGGAAGG |
|  | Reverse primer | AAACTGCACCACAGAACCATCCTG |
| MBNL1 | Forward primer | CAGCAGCCGCCTTTAATCCCTATC |
|  | Reverse primer | CACCCGGATTCCCTGTAACCAAC |
| EFNA5 | Forward primer | AACCAGCAGATGACACCGTACATG |
|  | Reverse primer | TCGCCAGGAGGAACAGTAGGATTG |
| HOPX | Forward primer | CACCACGCTGTGCCTCATCG |
|  | Reverse primer | GTCTGTGACGGATCTGCACTCTG |
| ZC3H12C | Forward primer | GGAAAGAGCAATCCCGACCTGATG |
|  | Reverse primer | TCGTCATAGCACACCACTCTCCTC |
| GAPDH | Forward primer | CAAGGTCATCCATGACAACTTTG |
|  | Reverse primer | TCCACCACCCTGTTGCTGTAG |
| N-cadherin | Forward primer | TGGACCATCACTCGGCTTA |
|  | Reverse primer | ACACTGGCAAACCTTCACG |
